# A study of Smad4, Smad6 and Smad7 in Surgically Resected Samples of Pancreatic Ductal Adenocarcinoma and Their Correlation with Clinicopathological Parameters and Patient Survival

**DOI:** 10.1186/1756-0500-4-560

**Published:** 2011-12-23

**Authors:** Puneet Singh, Radhika Srinivasan, Jai Dev Wig, Bishan Das Radotra

**Affiliations:** 1 Department of General Surgery, Postgraduate Institute of Medical Education and Research, Chandigarh, India; 2 Department of Cytology and Gynaecological Pathology, Postgraduate Institute of Medical Education and Research, Chandigarh, India; 3 Department of Histopathology, Postgraduate Institute of Medical Education and Research, Chandigarh, India

**Keywords:** Pancreas, pancreatic adenocarcinoma, Smad4, Smad6, Smad7, clinicopathological parameters, prognosis

## Abstract

**Background:**

Smad4 is the common mediator of the tumor suppressive functions of TGF-beta. Smad6 and Smad7 are the antagonists of the TGF-beta pathway. This study investigates the differential protein expressions of Smad4, Smad6 and Smad7 in tumor as compared to normal tissue of pancreatic ductal adenocarcinoma (PDAC) and compares them with clinicopathological parameters and patient survival.

**Results:**

There was a significant difference in protein expressions of Smad4 (p = 0.0001), Smad6 (p = 0.0015) and Smad7 (p = 0.0005) protein in tumor as compared to paired normal samples. Loss of Smad7 expression correlated significantly with tumor size (r = 0.421, p < 0.036) and margin status (r = 0.431; p < .032). Patients with moderate to high Smad4 protein expression had a better survival (median survival = 14.600 ± 2.112 months) than patients with absent or weak Smad4 protein expression (median survival = 7.150 ± 0.662). In addition, advanced disease stage correlated significantly with poor prognosis.

**Conclusion:**

Loss of Smad4 significantly correlated with poor survival of PDAC patients. In the cases where Smad4 is expressed, Smad6 inhibition is possibly a novel mechanism for Smad4 inactivation. Smad7 has a role in pathobiology of PDAC. Further investigation in the roles of Smad6 and Smad7 would help in the identification of novel therapeutic targets for PDAC.

## Background

Smad proteins are a family of intracellular mediators of the transforming growth factor beta (TGF-β) family of cytokines. On ligand binding, TGF-β Receptor II (TβRII) becomes constitutively active, heterodimerizes with TGF-β Receptor I (TβRI) and transphosphorylates its GS domain resulting in its activation [1,2]. Once activated, TβRI phosphorylates a class of molecules known as receptor-regulated Smads (R-Smads), Smad2 and Smad3, at an SSXS motif at their C-terminal end [3]. Active, phosphorylated R-Smads heterodimerize with common-Smad (Co-Smad), Smad4, translocate to the nucleus and regulate gene expression [4,5]. A third class of Smad proteins, the inhibitory Smads (I-Smads), Smad6 and Smad7 act as negative regulators and act by blocking R-Smads' interaction with TβRI, phosphorylation by TβRI or heterodimerization with Smad4 [6,7].

Smad4 is being explored as one of the major molecular markers in pancreatic ductal adenocarcinoma (PDAC) (as reviewed by [8]). Although lost in many cancers, loss of Smad4 is more sensitive and specific to pancreatic cancer [9]. Studies have shown *SMAD4 *gene to be inactivated in 55% of pancreatic cancers [10-12]. The inactivation of *SMAD4 *gene occurs either by deletion of both alleles (35%) or by intra-genic mutation in one allele coupled with the loss of the other allele (20%) [13]. A number of studies demonstrate the role of Smad4 in pancreatic ductal adenocarcinoma, but only a few studies have explored the roles of inhibitory Smads, Smad6 and Smad7 in this disease (as reviewed by [14]).

In the present study, we examined the differential protein expressions of Smad4, Smad6 and Smad7 in surgically resected samples of paired tumor tissue of pancreatic ductal adenocarcinoma versus adjacent normal tissue. A combinatorial expression of these three Smads was evaluated to gain an insight into how they influenced one another in pancreatic ductal adenocarcinoma as compared to normal pancreas. Influence of the expression levels of these proteins on clinicopathological parameters and patient survival was studied.

## Methods

### Patients

The study was conducted after obtaining a formal approval from the Ethics Committee of the Postgraduate Institute of Medical Education and Research. Informed oral consent was obtained from each patient for participation in the study.

Twenty-five prospective cases of histopathologically proven pancreatic ductal adenocarcinoma, collected over a period of 36 months at the Department of General Surgery, Postgraduate Institute of Medical Education and Research, Chandigarh, India, were included in our study. Out of these 25 subjects, 13 were males and 12 were females. The mean age of the patients was 54.6 years, with two patients below 40 and one as young as 28. Follow-up data was collected for all patients. Most PDAC tumor samples were either highly differentiated (13) or moderately differentiated (11), with just one case poorly differentiated. The patients were staged according to the Tumor, Node and Metastasis (TNM) classification of the International Union against Cancer [15]. Clinicopathological and outcome data is summarized in Table 1.

**Table 1:** Clinical profile of patients with pancreatic ductal adenocarcinoma (n = 25)

Clinical variable	Groups	No. of patients
Age (range 28-75)	< 50	5(20%)
	
	≥ 50	20(80%)

Sex	Male	13 (52%)
	
	Female	12 (48%)

Stage	I	3 (12%)
	
	II	7 (28%)
	
	III	14 (64%)
	
	IV	1 (4%)

Grade	Well differentiated	13 (52%)
	
	Moderately/poorly differentiated	12 (48%)

Tumor size	< 3 cm	19 (76%)
	
	≥3 cm	6 (24%)

Margin status	Positive	3 (12%)
	
	Negative	22 (88%)

Lymph Node status	Positive	6 (24%)
	
	Negative	19 (76%)

### Tissue Sample

Surgically resected samples were collected and tumor was confirmed by performing hematoxylin and eosin (H&E) staining on frozen sections taken on autoclaved glass slides. Similarly, the presence of normal pathology in the adjacent normal tissues was also confirmed. Initially, samples from a total of 32 consecutive patients were collected, out of which 25 samples that showed tumor tissue in more than 90% of the area of the section were included for study. Part of the samples to be used for immunohistochemistry were formalin fixed, and part of them were snap frozen and stored at - 80°C for further molecular analysis.

### Clinicopathological Data

Clinical and pathological data were obtained from the patients' medical records. Clinical and pathological variables included age, gender, tumor size, margin status, stage, grade, and survival.

### Immunohistochemistry

Immunohistochemical labeling was done on 4 μm tissue sections mounted on slides coated with poly-L-lysine (Sigma, St. Louis, Missouri, USA) using the routine streptavidin - biotin immunoperoxidase technique. Sections were deparaffinised in xylene, rehydrated through a series of graded alcohol to distilled water and microwaved in buffered sodium citrate. Endogenous peroxidase was blocked by incubating in hydrogen peroxidase with methanol followed by overnight incubation with monoclonal antibodies, anti-Smad4 (clone B-8), anti-Smad6 (clone H-150) and anti-Smad7 (clone H-79), obtained from Santa Cruz Biotechnology Inc., Santa Cruz, California, USA. Novastatin Universal Detection kit (Ready to use, Novacastra Laboratories Ltd., Newcastle, UK) containing biotinylated secondary antibody was applied and staining was visualised using 3', 3'- Diaminobenzidinetetrahydrochloride (Sigma Chemical Co., St. Louis, USA) solution as the chromogen. The sections were counterstained in Mayer's haematoxylin, rinsed in water, and mounted in Di-N-Butyle Phthalate in Xylene. The brown product obtained was visualized and scored by light microscopy. Antigen retrieval conditions and the antibody dilutions used are summarized in Table 2.

**Table 2:** Antibodies used in the study

Antibody	Clone*	Antigen retrieval	Primary antibody incubation	Concentrations used
anti-Smad4	Clone B-8	Pressure cooker for 20 min	2 h at room temperature	2 μg/ml (1:100)

anti-Smad6	Clone H-150	Microwaving:3 Cycles 3 min +1 Cycle 1 min	Overnight at 4°C	4 μg/ml (1:50)

anti-Smad7	Clone H-79	Microwaving: 3 Cycles 3 min +1 Cycle 1 min	Overnight at 4°C	4 μg/ml (1:50)

*All antibodies were obtained from Santa Cruz Biotechnology Inc., CA., USA.

### Immunohistochemical Evaluation

Immunohistochemical scoring was done independently by two senior cytopathologists (BDR and RS) and only samples with complete concordance in staining and histopathology were included in the study. The slides were scored as follows: 0 (no staining), 1+ (weak staining), 2+ (moderate staining), and 3+ (strong staining), a scoring system previously described by Hua *et al *[16]. Paired adjacent normal tissue samples served as positive controls for each of the cases. There was a complete concordance in all the cases except one, where high and moderate expression of Smad4 for the same normal tissue sample was respectively reported. Re-evaluation, however, eliminated the discrepancy.

### Statistical Methods

Fisher's exact test was used to compare Smad4, Smad6 and Smad7 protein expression in normal and tumor tissue. Spearman rank correlation test was used to correlate Smad4, Smad6 and Smad7 protein expression in tumor tissue with clinicopathological parameters. Kaplan-Meier survival analysis was used to analyse the influence of Smad4, Smad6 and Smad7 protein expression in tumor tissue and clinicopathological parameters on survival. A probability value of less than 0.05 was considered to be significant.

## Results

### Immunohistochemical expression of Smad4, Smad6 and Smad7

**Figure 1:**
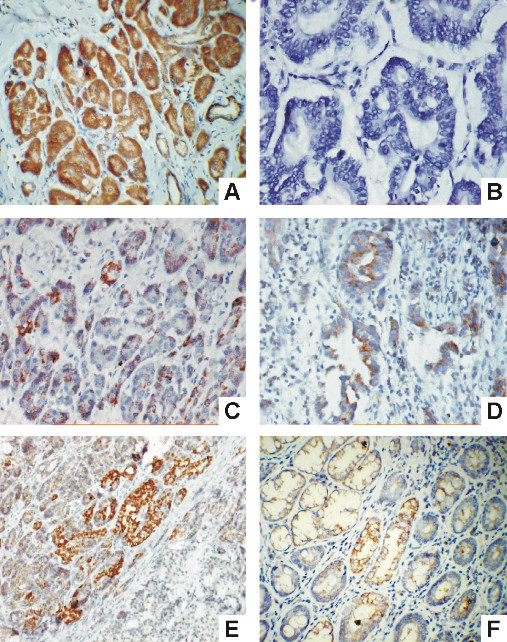
**Immunohistochemistry for Smads in paired tumor and normal pancreatic tissues**. Upper panel shows the normal pancreatic tissue and the down panel shows the corresponding tumor tissue. Normal pancreas with strong focal and nuclear positivity (a. ×100) and tumor negative for Smad4 (b. ×400). Normal pancreas with strong diffuse cytoplasmic positivity (c. ×100) and tumor with moderate focal cytoplasmic positivity for Smad6 (d. ×200). Normal pancreas with strong diffuse cytoplasmic positivity (e. ×100) and tumor negative for Smad7 protein (f. ×200). (streptavidin- biotin immunoperoxidase).

**Figure 2:**
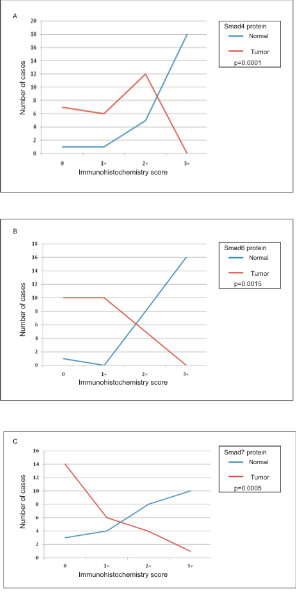
**Frequency of protein expression in normal pancreas and pancreatic ductal adenocarcinomas for Smad4, Smad6 and Smad7, respectively**. The x-axis represents the immunohistochemistry score and y-axis represents the number of cases.

Protein levels of all three Smads, Smad4, Smad6 and Smad7 were evaluated in paired normal pancreatic tissues and tumor samples of pancreatic ductal adenocarcinoma (Figure 1; Table 3). A comparison of the protein levels between normal and tumor tissues for each of the three Smads is shown in Figure 2.

**Table 3:** Immunohistochemical expression of Smad4, Smad6 & Smad7 in paired samples of Normal pancreas and Pancreatic ductal adenocarcinoma (n = 25)

IHC Score	Smad4		Smad6		Smad7	
	
	Normal	Tumor	Normal	Tumor	Normal	Tumor
0	1(4%)	10(40%)	1(4%)	7(28%)	3(12%)	14(56%)

1+	0(0%)	10(40%)	1(4%)	6(24%)	4(16%)	6(24%)

2+	8(32%)	5(20%)	5(20%)	12(48%)	8(32%)	4(16%)

3+	16(64%)	0(0%)	18(72%)	0(0%)	10(40%)	1(4%)

### Smad4

Smad4 showed cytoplasmic as well as nuclear staining, which was both diffuse and focal (Figure 1a, b). Most normal tissue showed strong to moderate Smad4 immunoreactivity (24/25, 96%), whereas most tumor tissue showed absent (10/25, 40%) or weak (10/25, 40%) immunoreactivity for Smad4. Strong to moderate expression was seen only in 20% (5/25) of tumor samples. The difference in Smad4 protein levels in tumor tissue as compared to normal pancreatic tissue was highly significant on Fisher's exact test (two tailed p value = 0.0001).

### Smad6

The protein immunoreactivity was predominantly cytoplasmic (Figure 1c, d). Here also, most of the normal tissues showed strong (18/25, 72%) to moderate (5/25, 20%) immunopositivity. However, A good number (12/25, 48%) of tumor tissues showed moderate levels of Smad6 protein expression, in contrast to Smad4 where most of the cases were weakly positive or absent. Complete loss of Smad6 expression was seen only in 28%(7/25) of tumor cases. The difference in Smad6 protein levels in tumor tissue as compared to normal pancreatic tissue was highly significant by Fisher's exact test (p = 0.0015).

### Smad7

The immunoreactivity was predominantly cytoplasmic, although occasional nuclear positivity was obtained in some normal pancreatic ducts (Figure 1e, f). Similar to Smad4, normal pancreatic tissue showed moderate to high levels of Smad7 expression in most of the samples (18/25, 72%), whereas, more than half of tumor tissue showed complete loss of protein expression (4/25, 56%), and another 24%(6/25) of cases showed low expression. The difference in the expression levels of Smad7 in normal pancreatic samples as compared to tumor samples was highly significant by Fisher's exact test (p = 0.0005).

### Co-expression of Smad4 with inhibitory Smads

In tumor samples, out of 15 Smad4 positive cases, 10 showed low and 5 showed moderate Smad4 protein expression. In four out of these five cases, either or both Smad6 and Smad7 were moderately co-expressed. Overall, out of 15 Smad4 positive cases, 14 cases showed either Smad6 or Smad7 expression, with almost all cases except one showing Smad6 expression (Table 4).

**Table 4:** Combinatorial expression of Smad4, Smad6 & Smad7 in 25 tumor samples of pancreatic ductal adenocarcinoma patients

	Positive cases			
	
	Smad6 (n = 18, 72.0%)	Smad7 (n = 11, 44.0%)	Smad6 or Smad7 (n = 21, 84.05)	Smad6 and Smad7 (n = 9, 36%)
**Smad4 positive cases (n = 15)**	14 (93.3%)	9 (60.0%)	15 (100%)	5 (55.5%)

**Smad4 negative cases (n = 10)**	4 (40.0%)	2 (20.0%)	9 (90.0%)	4 (44.5%)

### Correlation of Smad4, Smad6 and Smad7 protein expression with clinocopathological parameters

A comparison of various clinicopathological parameters with Smad4, Smad6 and Smad7 protein expressions using Spearman correlation was done (Table 5). Absent/low Smad7 protein expression showed a significant positive correlation with tumour size (r = 0.421, p < .036) and margin status (r = 0.431; p < .032).

**Table 5:** Correlations of the expression of Smad4, Smad6 and Smad7 with clinicopathological parameters in 25 patients of pancreatic ductal adenocarcinoma

Parameters	Groups	No.(n)	Loss/low expression of Smad4 (%)	P value (2-tailed)	Loss/low expression of Smad 6 (%)	P value	Loss/low expression of Smad7(%)	P value
**Age**	< 50	5	4 (16%)	1.000	4 (16%)	0.244	4 (16%)	1.000
	
	≥ 50	20	16 (40%)		10 (40%)		16 (64%)	

**Sex**	Male	13	10 (40%)	0.704	7(28%)	0.830	10(40%)	0.704
	
	Female	12	10 (40%)		7 (28%)		10 (40%)	

**Grade**	G1	13	11 (44%)	0.567	8 (32%)	0.580	9 (36%)	0.175
	
	G2	12	9 (36%)		6 (24%)		11 (44%)	

**Stage**	I+II	10	7 (28%)	0.328	5 (20%)	0.639	8 (32%)	1.000
	
	III+IV	15	13 (52%)		9 (36%)		12 (48%)	

**LN status**	Negative	19	15 (60%)	0.824	9 (36%)	0.132	15 (60%)	0.824
	
	Positive	6	5 (20%)		5 (20%)		5(20%)	

**Tumor size**	< 3 cm	19	16 (64%)	0.370	11(44%)	0.747	17 (68%)	0.036*
	
	≥3 cm	6	4 (16%)		3 (12%)		3 (12%)	

**Margin status**	Positive	3	2 (8%)	0.558	1(4%)	0.420	1 (4%)	0.032*
	
	Negative	22	18 (72%)		13 (52%)		19 (76%)	

*Significant values

### Univariate analysis for survival

**Figure 3:**
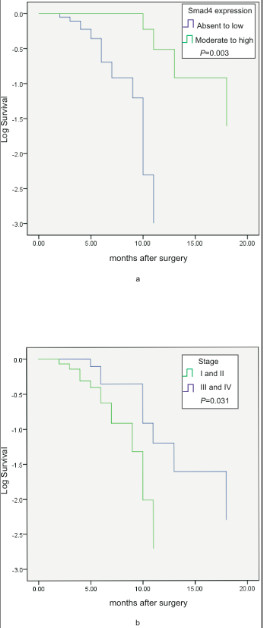
**Kaplan-Meier plot for disease specific survival: according to Smad4 protein expression (a.) and according to Stage of pancreatic ductal adenocarcinoma (early: I + II; advanced: III + IV) (b.)**.

The mean (± SEM) survival of patients was 8.64 ± 4.5 months and the median survival was 9 months. 20% of patients survived over one year. Kaplan-Meier analysis for survival demonstrated that patients with moderate to high Smad4 protein expression had a better survival (median survival = 14.600 ± 2.112 months) than patients with absent or weak Smad4 protein expression (median survival = 7.150 ± 0.662) [Log Rank (Mantel-Cox) Chi Square 9.116, significance .003] (Figure 3, Table 6). Even on adjusting individually for the stage, tumor size, grade and margin status of PDAC tumor samples, moderate to high Smad4 protein expression positively influences survival significantly [Log Rank (Mantel-Cox) Chi-Square 8.250, significance .004; Log Rank (Mantel-Cox) Chi-Square 9.772, significance .002; Log Rank (Mantel-Cox) Chi-Square 9.377, significance .002; Log Rank (Mantel-Cox) Chi-Square 8.524, significance .004]. Smad 6 and Smad 7 protein expression did not influence survival. Stage I & II patients showed a longer survival (median survival 10 ± 2.066 months) as compared to those in Stage III & IV (median survival 7 ± .949 months) [Log Rank (Mantel-Cox) Chi-Square 4.644, significance .031] (Figure 3, Table 6).

**Table 6:** Summary of results of survival analysis by Kaplan-Meier test

Variables	Groups	No. of patients	Median	95% *Confidence Interval *(*CI*)		*Significance*
					
				Lower	Upper	
**Age**	< 50	5	5.000 ± 2.191	0.706	9.294	0.211
	
	≥ 50	20	9.000 ± 2.225	4.639	13.361	

**Sex**	Male	13	10.000 ± 1.754	6.562	13.438	0.127
	
	Female	12	6.000 ± 1.732	2.605	9.395	

**Grade**	G1	13	7.000 ± 1.198	4.651	9.349	0.228
	
	G2	12	10.000 ± 1.125	7.795	12.205	

**Stage**	I+II	10	10.000 ± 2.066	5.951	14.049	0.031*
	
	III+IV	15	7.000 ± 0.949	5.141	8.859	

**Tumor size**	< 3 cm	19	9.000 ± 0.933	7.172	10.828	0.842
	
	≥ 3 cm	6	6.000	-	-	

**Margin status**	Negative	22	9.000 ± 1.038	6.966	11.034	0.999
	
	Positive	3	6.000	-	-	

**pSmad4**	Absent/Low	20	6.000 ± 0.745	4.539	7.461	0.003*
	
	Moderate/High	5	13.000 ± 2.191	8.706	17.294	

**pSmad6**	Absent/Low	14	7.000 ± 0.926	5.185	8.815	0.141
	
	Moderate/High	11	10.000 ± 1.477	7.105	12.895	

**pSmad7**	Absent/Low	20	7.000 ± 2.236	2.617	11.383	0.633
	
	Moderate/High	5	10.000 ± 3.286	3.559	16.441	

*The significant values are highlighted.

## Discussion

Smad4 is the common mediator of the tumor suppressive functions of TGF-beta. Smad6 and Smad7 are antagonists of the TGF-beta pathway. In this work, we further establish the role of Smad4 as a potential prognostic marker for pancreatic ductal adenocarcinoma. We also identified different roles for Smad6 and Smad7 in influencing pancreatic cancer biology.

In this study, Smad4 was expressed in most of the normal samples (96%) but lost in 40% of tumor samples. In tumor samples, even where it was expressed, there was weak expression in the majority of cases. Kaplan-Meier analysis for survival demonstrated that patients with moderate Smad4 protein expression had a better survival than patients with weak or negative Smad4 protein expression. Despite one report of Smad4 expression to be inversely related to survival in surgically resected pancreatic ductal adenocarcinoma patients [17], there is growing evidence for the correlation of Smad4 status to patient survival in this disease [16,18]. One study also correlated the Smad4 expression with the pattern of disease progression (local v distant dominant) and proposed to further explore its role as a predictive biomarker for personalized treatment strategies [19]. Our observations further adds to preexisting data and establish Smad4 as a potential prognostic marker for pancreatic ductal adenocarcinoma. However, a recent meta-analysis analyzing 5 studies evaluating Smad4 could not find any significant overall association between Smad4 expression and survival [20]. This indicates difficulty in making a reliable conclusion regarding the relative prognostic value of immunohistochemical markers when analyzed in a limited patient series.

For Smad6 and Smad7, although occasional samples showed nuclear staining, cytoplasmic staining was predominant. Previous reports have shown that while Smad7 appears to reside predominantly in the nucleus at basal state, it translocates to the cytoplasm upon TGF-β stimulation [21]. The cytoplasmic staining of Smad6 and Smad7 in most samples implies that these two inhibitory Smads were in their activated states in most tumor samples.

There are just two reports on Smad6 expression in pancreatic cancer till date. One of them conducted in pancreatic cancer cell line, found Smad6 and Smad7 levels to be elevated in pancreatic cancer [22]. The second study, conducted on patient samples, contradicts this and demonstrates that the increased expressions of either Smad6 or Smad7 are infrequent in tumor compared to normal samples [23]. Our study goes a step further, and shows that Smad6 as well as Smad7 are lost in tumor as compared to normal samples. We showed a loss of expression of Smad6 in 28% tumor samples as compared to 8% loss in normal samples. In cases where Smad6 was expressed, the expression was mostly moderate to high. Its cytoplasmic staining, along with its co expression with Smad4 in 14 out of 15 Smad4 positive cases suggests that Smad6 can be one of the possible inhibitory mechanisms for Smad4 inactivation. Thus, in pancreatic ductal adenocarcinoma cases where Smad4 itself is not lost, we illustrate a novel mechanism for its inhibition. Our study, for the first time ascribes Smad6 a role in pancreatic cancer biology, which can be further explored for the development of novel therapeutic target.

Previous studies have shown Smad7 overexpression in pancreatic cancer cell lines [22-24]. Similar to our study, few other studies have shown a loss of Smad7 in patient samples [24,25]. This difference in expression in cell lines as compared to tissue samples might be because of a possible reversal of phenotype in artificial tissue culture systems. In our study, loss of Smad7 expression surpassed that of Smad4 and was absent in 56% of tumor samples, which is quite close to what has been reported by Guo et al [24]. Amongst clinicopathological parameters, loss of Smad7 significantly correlated with both tumor size as well as margin status. On similar lines, Wang et al showed a significant correlation between the low Smad7 expression and lymph node metastasis [26]. These observations, put together, indicate a role for Smad7 in the aggressiveness of this disease. In fact, different studies have isolated different molecules, like KLF11, retinoblastoma, thioredoxin, which are involved in Smad7 dependent aggressiveness of pancreatic cancer [23,27,28]. However, unlike Wang et al, we did not find a significant correlation between loss of Smad7 and patient survival.

The smaller sample size in the study is acknowledged, the reasons being i) choice of prospective samples for the study, ii) low incidence of pancreatic cancer in Indian population: 0.5-2.4 per 100000 men and 0.2-1.8 per 100000 women [29], iii) limited time for the collection of patient samples, iv) exclusion of archival samples due to poor and unreliable staining.

## Conclusions

The present study strongly substantiates the previous reports in further establishing the role of Smad4 as a prognostic marker. It also suggests that a further exploration into the newly found roles for Smad6 and Smad7 in PDAC biology, with a larger sample size, may help discern some novel therapeutic targets for this disease, subsequently contributing to the improvement in therapeutic strategies and better disease management for PDAC patients.

## Competing interests

The authors declare that they have no competing interests.

## Authors' contributions

Author PS conducted the IHC experiments, compiled the data and drafted the manuscript. RS and BDR did IHC evaluation. JDW applied the biostatistics. RS, JDW and BDS helped in the design of study. All authors read and approved the final manuscript.
